# Fermentation with Aquilariae Lignum Enhances the Anti-Diabetic Activity of Green Tea in Type II Diabetic db/db Mouse

**DOI:** 10.3390/nu6093536

**Published:** 2014-09-09

**Authors:** Su Jin Kang, Ji Eun Lee, Eun Kyung Lee, Dae Hwa Jung, Chang Hyun Song, Soo Jin Park, Seong Hun Choi, Chang Hyun Han, Sae Kwang Ku, Young Joon Lee

**Affiliations:** 1 The Medical Research Center for Globalization of Herbal Medicine, Daegu Haany University, Gyeongsan 712-715, Korea; E-Mails: vegonia1@hanmail.net (S.J.K.); jelee910@naver.com (J.E.L.); kkong0305@hanmail.net (E.K.L.); dvmsong@hotmail.com (C.H.S.); sjp124@gmail.com (S.J.P.); 2 Department of Preventive Medicine, College of Korean Medicine, Deagu Haany University, Gyeongsan 712-715, Korea; 3 Department of Histology and Anatomy, College of Korean Medicine, Daegu Haany University, Gyeongsan 712-715, Korea; E-Mail: ck0190@hanmail.net; 4 HaniBio, Gyeongsan 712-260, Korea; E-Mail: jdh8024@hanmail.net; 5 Department of Medical History & Literature Group, Korea Institute of Oriental Medicine, Daejeon 305-811, Korea; E-Mail: chhan@kiom.re.kr

**Keywords:** BKS.Cg-+Leprdb/+Leprdb/OlaHsd (db/db) mice, obese, diabetes, fermented green tea with Aquilariae Lignum, synergistic

## Abstract

The major components of tea may be significantly influenced according to the type of fermentation, and consequently the effects of different teas will differ. We examined whether green tea fermented with Aquilariae Lignum (fGT) shows a stronger anti-diabetic effect than unfermented green tea (GT) on mice with type 2 diabetes. To evaluate the anti-obesity effect of fGT, we assessed body weight, fecal excretion, serum leptin levels, exocrine pancreatic zymogen granule contents, and periovarian fat weight and adiponectin contents. Blood glucose levels, pancreatic weight, and numbers of pancreatic islet insulin- and glucagon-producing cells were determined to evaluate anti-hypoglycemic effects, while total cholesterol, triglyceride, and low- and high-density lipoprotein levels were determined to evaluate anti-hyperlipidemic effects. The antioxidant effect of fGT was detected by measuring malondialdehyde and glutathione contents and the activities of catalase and superoxide dismutase. fGT showed anti-obesity, anti-hypoglycemic, anti-hyperlipidemia, and antioxidant effects. Additionally, fGT exerted stronger anti-diabetic effects compared with GT. Collectively, these results suggested that fGT fermented with the appropriate amounts of Aquilariae Lignum (49:1) has a stronger effect compared with GT. Thus, fGT is a promising and potent new therapeutic agent for type 2 diabetes.

## 1. Introduction

Type 2 diabetes, the most common form of diabetes, is a lifestyle-related diabetes or non-insulin-dependent diabetes and is a fast-growing and potentially life-threatening disease. Epidemiological and experimental evidence strongly indicates that hyperglycemia is the major cause of complications such as retinopathy, neuropathy, stroke, and amputations [1]. Many studies suggest that effective maintenance of blood glucose is a key approach to preventing or reversing diabetes complications and improving quality of life in patients with diabetes [2].

Insulin resistance, which characterizes type 2 diabetes and is a key factor in its development, can cause hyperglycemia, dyslipidemia, or hepatic steatosis. The liver is the major organ controlling glucose and lipid homeostasis. Notably, hepatic insulin resistance can result in hyperglycemia through reduced glycogen synthesis and storage and a failure to suppress glucose production and release into the blood. Additionally, insulin resistance is closely related to hepatic steatosis; however, it is not known whether lipid overaccumulation in the liver results from or leads to insulin resistance [3]. Reactive oxygen species (ROS) generated as a result of hyperglycemia or hyperlipidemia are one of the major causes of insulin resistance [4], while antioxidants such as quercetin, ascorbic acid, and β-carotene can improve antioxidant defense in diabetic rats [5].

The progression of type 2 diabetes is characterized primarily by a defect in β-cell function and worsening of insulin resistance. Once hyperglycemia becomes apparent, β-cell function gradually deteriorates, and insulin resistance is aggravated. Glucose-stimulated insulin secretion is impaired further, and degranulation of β-cells becomes evident, frequently with a reduction in the number of β-cells [6]. Most of the increase in β-cell mass with insulin resistance is probably due to an increase in the β-cell number, but β-cell hypertrophy may also contribute. Regulation of β-cell mass is a dynamic process, allowing control of glycemia within a narrow physiological range [7]. Numerous patients with obesity-associated insulin resistance do not have diabetes, as their capacity for β-cell compensation is maintained. Over time, 15%–20% of these individuals develop diabetes when their β-cells fail to maintain their compensatory ability [7]. Loss of β-cell function is therefore pivotal in defining the risk and development of type 2 diabetes, and one potential method to prevent and treat diabetes is the enhancement of β-cells.

Obesity is strongly related to the etiology of type 2 diabetes [8] and is responsible for the changes in the physiological function of adipose tissue. Adipose tissue can release increased amounts of non-esterified fatty acids, glycerol, hormones, pro-inflammatory cytokines, and other factors, leading to insulin resistance, chronic inflammation, and altered secretion of adipokines [9]. Adipokines may regulate insulin sensitivity by activating multiple signaling pathways after phosphorylation of the insulin receptor and several other molecules in type 2 diabetes [10].

The tea plant (*Camellia sinensis* L.) was popularly consumed by ancient cultures for its medicinal properties [11]. Tea is classified into unfermented (green tea (GT)), semifermented (oolong tea), and fermented (black and pu-erh or red) forms [12]. The components of the various forms of tea show different bioactivities. Tea has antioxidant [13], pro-immune [14], anti-atherosclerotic [15], antihypertension [16], anti-infectious disease [17], and antidiabetic properties [18].

Aquilariae Lignum is the stem parts of *Aquilaria agallocha* Roxb (Thymelaceae) that contains essential oils. The chemical components of Aquilariae Lignum include benzylacetone, p-methoxybenzylacetone, hydrocinnamic acid, agarospirol, agarofuran, and dihydroagarofuran [19]. Aquilariae Lignum has been used traditionally in aromatherapies for various purposes, including anti-allergic [20], analgesic [21], and anxiolytic [22] effects. As a potent ROS scavenger, it is expected to have a therapeutic role in diabetic mellitus. Our screening test showed that an aqueous extract of green tea fermented with Aquilariae Lignum (49 g/1 g, fGT) has potent hypoglycemic, hypolipidemic, and anti-obesity effects on diabetic db/db mice.

The components of different forms of tea (unfermented, semifermented, and fermented) differ in bioactivity [23]. For this reason, we examined whether GT fermented with Aquilariae Lignum shows a stronger anti-diabetic effect than does unfermented GT on obese db/db mice with type 2 diabetes. Our results indicate that fGT and Aquilariae Lignum have a positive synergistic effect in db/db mice.

## 2. Experimental Section

### 2.1. Animals and Husbandry

Ten normoglycemic intact specific pathogen-free female C57BL/6NCrljOri mice (6-week old upon receipt; OrientBio, Seungnam, Korea), and 55 female genetically diabetic specific pathogen-free BKS.Cg-+Leprdb/+Leprdb/OlaHsd, db/db mice (6-week old upon receipt; Harlan, Indianapolis, IN, USA) were used after acclimatization for 14 days. Animals were allocated four per polycarbonate cage in a temperature (20 °C–25 °C) and humidity (40%–45%) controlled room. Light: dark cycle was 12 h:12 h, and standard rodent chow (Samyang, Seoul, Korea) and water were supplied free to access. Animals were divided into 7 groups based on body weight at 14 days after acclimatization as follows, normoglycemic intact mice (mean 18.73 ± 0.76 g, ranged in 17.60~19.90 g) and hyperglycemic obese mice (39.83 ± 1.83 g, ranged in 36.60~44.30 g), respectively. All laboratory animals were treated according to the national regulations of the usage and welfare of laboratory animals, and approved by the Institutional Animal Care and Use Committee in Daegu Haany University (Gyeongsan, Gyeongbuk, Korea) prior to animal experiment (Approval No. DHU2013-041, approved on 3 July 2013). In present study, 400 mg/kg fGT extracts were selected based on our previous preliminary results [24], and 200 and 100 mg/kg were determined as middle and the lowest dosages using common ratio 2 on db/db mice. Metformin hydrochloride (Wako, Osaka, Japan) were used as reference recommendation drug. Metformin 250 mg/kg, GT 400 mg/kg, or fGT 400, 200 and 100 mg/kg were orally administered, dissolved in distilled water, once a day for 84 days from 14 days of acclimatization, in a volume of 10 mL/kg. In vehicle and db control mice, only equal volumes of distilled water were orally administered, instead of aqueous extracts of fGT and GT or metformin, respectively.

### 2.2. Preparations of Test Substances

Light brown solution of fGT and greenish brown solution of GT were prepared by sponsor (ChuiWoon HyangDang, Seongju, Korea). One year aged fGT were used in this study. The process for making fGT was as follows. Mixtures of dried green tea leaves and Aquilariae Lignum powder (49 g/1 g) were wet-fermented for 12 h at 60 °C, steamed for 30 s at 100 °C, and dried for 1 week at 15 °C.

The steamed mixtures were cooled and additionally dried at 15 °C for 3 days. Each of fGT or dried GT (28 g) were boiled at 100 °C for 6 h and then cooled for additional 6 h in 1 L of pure water, respectively. Aqueous solutions were completely lyophilized (Operon FDB-5503, Kimpo, Korea). Total 5.40 g of fGT (yield = 19.29%) and 7.28 g of GT (yield = 26.00%) were acquired. Lyophilized fGT and GT aqueous extracts were stored at −20 °C in a refrigerator to protect from light and humidity until used.

### 2.3. Changes in Body Weight

Changes of body weight were measured once a day for 84 days, from one day before initiation of administration using an automatic electronic balance (Precisa Instrument, Zuerich, Switzland). At initiation of administration and at a termination, all experimental animals were overnight fasted (water was not; about 18 h) to reduce the differences from feeding. In addition, body weight gains were calculated as follows:

Body weight gains (g) = Body weight at a termination − body weight at initiation of administration (From Day 0 to Day 84 of test article administration)
(1)

### 2.4. Food Consumption Measurements

All mice were housed in individual cages containing 150 g of food. Unconsumed food was weighed 24 h after it had been supplied using an automatic electronic balance (Precisa Instruments, Zurich, Switzerland). These are regarded as individual daily food consumption of mice (g/24 h/mouse). These measurements were conducted three times during administration, at 28, 63 and 83 days after first administration, respectively.

### 2.5. Water Consumption Measurements

All mice were allocated in individual cages contained 250 mL of water, and reminder volumes of supplied water were measured at 24 h after water supply using a measuring cylinder (Pyrex, Corning, NY, USA). These are regarded as individual daily water consumption of mice (mL/24 h/mouse). These measurements were conducted three times during administration, at 28, 63 and 83 days after first administration, respectively.

### 2.6. Fecal Excretion Measurements

The excreted fecal pellets of individual mice during 24 h were collected three times during administration, at 28, 63 and 83 days after first administration, and the total excreted fecal pellet weights were measured using an automatic electronic balance (Precisa Instrument, Zuerich, Switzland). These are regarded as individual fecal excretion of mice (g/24 h/mouse).

### 2.7. Urine Excretion Measurements

The excreted urines of individual mice during 24 h were collected at 28, 63 and 83 days after first administration, and the total excreted urine volumes were measured using a measuring cylinder (Pyrex, Corning, NY, USA). These are regarded as individual urine excretion of mice (mL/24 h/mouse).

### 2.8. Organ Weight Measurements

At sacrifice, the weights of liver, pancreas, left kidney and left periovarian fat pads were measured at g levels, individually, and to reduce the differences from individual body weight, the relative weight (% of body weight) were also calculated using body weight at sacrifice and absolute weight as follows:

Relative organ weight (%) = (Absolute organ weight/Body weight at sacrifice) × 100
(2)

### 2.9. Serum Biochemistry

At 28 days after treatment, bloods were collected from vena cava, and collected bloods were deposited into NaF glucose vacuum tubes (Becton Dickinson, Franklin Lakes, NJ, USA) or clotting activated serum tubes, separately. And then, centrifuged at 15,000 RPM for 10 min under room temperature for separating the plasma to blood glucose level measurements, and the serum to aminotransferase (AST), alanine aminotransferase (ALT), blood urea nitrogen (BUN), creatinine, total cholesterol (TC), total triglyceride (TG), low density lipoprotein (LDL) and high density lipoprotein (HDL) measurement. Blood glucose levels, and serum AST, ALT, BUN, creatinine, TC and TG levels were measured using automated blood analyzer (Hemagen Analyst, Hemagen Diagnostic, Columbia, MD, USA), and serum HDL and LDL were also detected by other typed using automated blood analyzer (AU400, Olympus, Tokyo, Japan), respectively.

### 2.10. Measurement of Serum Leptin and Adiponectin Levels

For detecting the serum adiponectin levels, serum was separated with general methods from collected blood. Serum leptin (Linco Research, St Charles, MO, USA) and adiponectin (Otsuka Pharm., Tokushima, Japan) levels were detected using a commercially available ELISA kits as previously [25].

### 2.11. Measurement of Adiponectin Contents in Periovarian Adipose Tissues

Adipose tissue adiponectin levels were determined by Western blot analysis. The removed epididymal adipose tissues were homogenized in PBS containing 0.5% sodium deoxycholate. Homogenates were incubated for 24 h at 37 °C. Aliquots of the tissue extracts (10 μg of protein) prepared in SDS sample buffer were incubated for 5 min at 100 °C. Denatured proteins were separated by SDS-PAGE and then transferred to polyvinylidene difluoride (PVDF) membranes (Bio-Rad Lab., Hercules, CA, USA). The membranes were incubated with anti-mouse adiponectin monoclonal antibody (Chemicon International, Temecula, CA, USA) for 12 h and then incubated with horseradish peroxidase-conjugated goat anti-mouse IgG antibody (DAKO Corp., Carpinteria, CA, USA) for 1 h. The membranes were exposed to X-ray film, and the adiponectin protein was thus visualized. The signals from X-ray film were quantified using DMI CCD image analyzer system (DMI, Daegu, Korea).

### 2.12. Liver Lipid Peroxidation and Antioxidant Defense Systems

After measurements of organ weight, the malondialdehyde (MDA) and glutathione (GSH) contents, catalase (CAT) and superoxide dismutases (SOD) enzyme activities in mouse liver tissues were assessed, respectively. Separated liver tissues were homogenized in ice-cold 0.01 M Tris-HCl (pH 7.4) as described by Kavutcu *et al*. [26]. The concentrations of liver lipid peroxidation were determined by estimating MDA using the thiobarbituric acid test at absorbance 525 nm, as nM of MDA/mg tissue [27]. Contents of total protein were measured by previous method [28] using bovine serum albumin (Invitrogen, Carlsbad, CA, USA). GSH contents were measured at absorbance 412 nm using 2-nitrobenzoic acid (Sigma-Aldrich, St. Louise, MO, USA) as μM/mg tissue [29]. Decomposition of H_2_O_2_ in the presence of catalase was followed at 240 nm [30]. Catalase activity was defined as the amount of enzyme required to decompose 1 nM of H_2_O_2_ per minute, at 25 °C and pH 7.8. Results were expressed as U/mg tissue. Measurements of SOD activities were made according to Sun *et al*. [31]. SOD activity was then measured at 560 nm by the degree of inhibition of this reaction, and was expressed as U/mg tissuen. One unit of SOD enzymatic activity is equal to the amount of enzyme that diminishes the initial absorbance of nitroblue tetrazolium by 50%.

### 2.13. Measurement of Lipid Compositions in the Feces

Lipid was extracted from feces, according to the method of Folch *et al*. [32]. The concentrations of fecal TC and TG measured by enzymatically using a commercial kit (Asan Pharmaceutical Co., Seoul, Korea) based on a modification of lipase-glycerol phosphate oxidase method [33].

### 2.14. Histopathology

Histopatholical profiles were examined in the left lateral lobes of liver, left kidney, splenic lobes of pancreas and periovarian fat pads. The tissue samples were fixed in 10% neutral buffered formalin, paraffin-embedded and then serially sectioned at 3–4 μm thickness. Representative section was stained with hematoxylin and eosin (H & E) for light microscopical examination. A portion of liver was further dehydrated in 30% sucrose solution, and cryo-sectioned for oil red stain [34]. The stains were observed in the restricted fields of view on a computer monitor, and examined using an automated image analysis process (iSolution FL ver 9.1, IMT i-solution Inc., Quebec city, Quebec, Canada). For further detailed histopathological changes, in the liver, mean diameter of hepatocyte (μm) was assessed in at least 10 hepatocytes of the H & E stain, and the regions of steatohepatitis were assessed as a percentage of lipid deposited regions in the hepatic parenchyma of oil red stain [34]. In the kidney, the vasodilated atrophic glomerulus was assessed as numbers/100 glomerulus. In the pancreas, the pancreatic islet was assessed as numbers/10 mm^2^ of pancreatic parenchyma, and its diameter (μm) was measured according to the established methods [35]. In addition, mean area occupied by zymogen granules was assessed as percentage of area of pancreatic parenchyma (mm^2^). In the periovarian fat pads, the thickness (mm) was measured, and mean diameter of periovarian white adipocytes (μm) was assessed in at least 10 adipocytes. All analyses were performed by a histopathologist blinded to groups.

### 2.15. Immunohistochemistry

Other serial section closed to the above H & E stain in the pancreas was immunostained for insulin and glucagon. The section was deparaffinized and rehydrated. Then, endogenous peroxidase activity was blocked by 0.3% H_2_O_2_ in methanol for 30 min, and non-specific binding of immunoglobulin was blocked by normal horse serum blocking solution (Vector Lab., Burlingame, CA, USA, dilution 1:100) for 1 h at room temperature in humidity chamber. Primary antiserum of guinea pig polyclonal insulin (DiaSorin, Stillwater, MN, USA, dilution: 1:2000) or rabbit polyclonal glucagon (DiaSorin, Stillwater, MN, USA, dilution: 1:2000) was treated for overnight at 4 °C in the humidity chamber. Next day, the sections were incubated with biotinylated universal secondary antibody (Vector Lab., dilution 1:50) for 1 h at room temperature, and then avidin-biotin-peroxidase reagents (Vectastain Elite ABC Kit, Vector Lab., dilution 1:50) according to ABC methods [36]. The section was reacted with peroxidase substrate kit (Vector Lab.) for 3 min at room temperature, and mounted with coverslips. All sections were rinsed with 0.01 M PBS 3 times between each step. The cells with density over 20% of the immunoactivities as compared with other naïve cells, were regarded as positive, and the positive cells were assessed as mean numbers in the area of pancreatic islets (mm^2^) using the automated image analysis process [37]. In addition, the ratio of insulin-positive to glucagon-positive cells was calculated. The histopathologist was blinded to the groups.

### 2.16. Statistical Analyses

All numerical values are expressed mean ± standard deviation (SD) of eight mice. Multiple comparison tests for different dose groups were conducted. Variance homogeneity was examined using the Levene test. If the Levene test indicated no significant deviations from variance homogeneity, the obtain data were analyzed by one way ANOVA test followed by least-significant differences (LSD) multi-comparison test to determine which pairs of group comparison were significantly different. In case of significant deviations from variance homogeneity were observed at Levene test, a non-parametric comparison test, Kruskal-Wallis H test was conducted. When a significant difference is observed in the Kruskal-Wallis H test, the Mann-Whitney U (MW) test was conducted to determine the specific pairs of group comparison, which are significantly different. Statistical analyses were conducted using SPSS for Windows (Release 14.0K, SPSS Inc., Armonk, NY, USA).

## 3. Results

### 3.1. Effects on Obesity

#### 3.1.1. Effects on the Body Weight Changes

**Figure 1:**
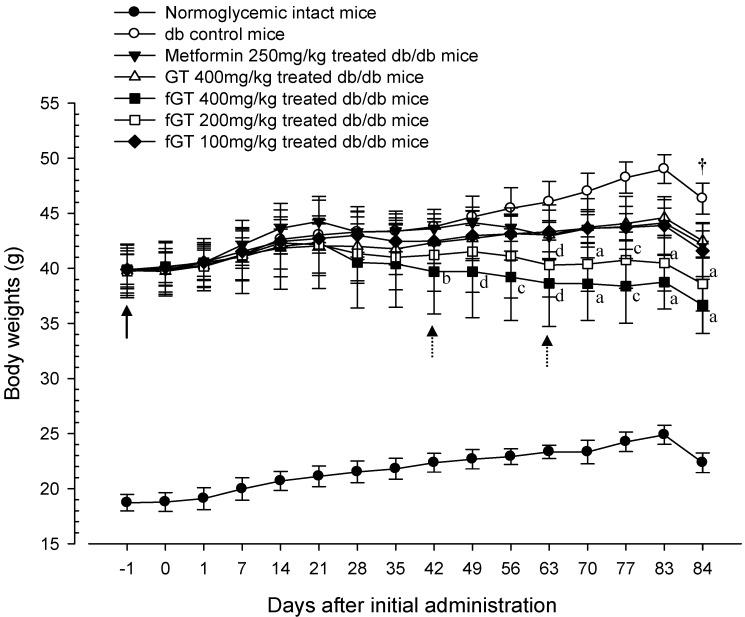
Body weight changes in intact Normoglycemic and db/db mice. Values are expressed as mean ± SD of eight mice. GT, Green tea aqueous lyophilized extracts; fGT, Aquilariae Lignum-fermented green tea aqueous lyophilized extracts. All animals were overnight fasted before sacrifice. ^a^ *p <* 0.01 and ^b^ *p <* 0.05 as compared with GT 400 mg/kg by LSD test; ^c^ *p <* 0.01 and ^d^ *p <* 0.05 as compared with GT 400 mg/kg by MW test.

**Figure 2:**
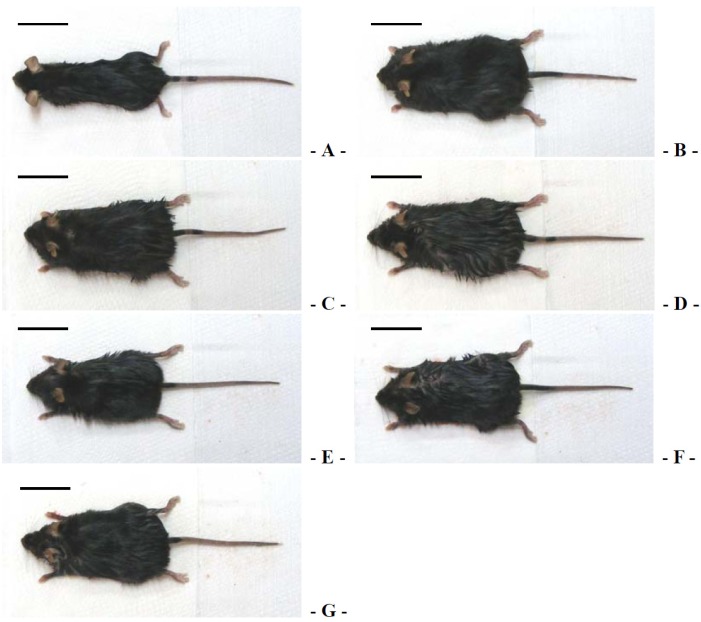
Representative gross body mass images, taken from intact normoglycemic or db/db Mice at sacrifice. (**A**) Intact control mouse; (**B**) db control mouse; (**C**) metformin 250 mg/kg treated db mouse; (**D**) GT 400 mg/kg treated db mouse; (**E**) fGT 400 mg/kg treated db mouse; (**F**) fGT 200 mg/kg treated db mouse; (**G**) fGT 100 mg/kg treated db mouse. GT, Green tea aqueous lyophilized extracts; fGT, Aquilariae Lignum-fermented green tea aqueous lyophilized extracts. Scale bar = 33 mm.

The db/db mice showed the marked increases of body mass and body weight from start of experiments. In addition, before treatment, compared with age-matched normoglycemic C57BL/6N intact mice, the body weight gains were significantly increased in db/db mice during 84 days of administration periods. In notice, the body weight in 400 and 200 mg/kg fGT extracts treated db/db groups were significantly decreased compared with the body weight of db control group (from day 28 and day 42, respectively, after the start of administration of test materials). In addition, body weight gains were significantly decreased in the 400 and 200 mg fGT extracts-treated db group compared with db control group (Figure 1 and Figure 2). In addition, the gains in body weight were more decreased in 400 and 200 mg/kg fGT extracts than 400 mg/kg GT extractl group from the day 42 and day 63. Compared with the intact control group, body-weight gains in the db control group were decreased by 78.60% during the 84-day drug administration period. They were changed by −64.24%, −56.39%, −154.62%, −121.02% and −76.42% in the 250 mg/kg metformin, 400 mg/kg GT extracts, 400 mg/kg, 200 mg/kg, and 100 mg/kg fGT groups, respectively, compared with the db control group.

#### 3.1.2. Effects on the Food and Water Consumption

As compared with intact control mice, db control mice showed an increase in food and water consumption at all measure times (28, 63 and 83 days, respectively) after initial administration. The treatment of 400 and 200 mg/kg fGT extract showed significant decreases of food and water consumptions as compared with GT extract 400 mg/kg treated db/db mice, at 63 and 83 days after initial administration, respectively. After 83 days, food consumption changed by −22.28%, −16.18%, −35.11%, −31.87%, and −15.48% in the 250 mg/kg metformin, 400 mg/kg GT extract, 400 mg/kg fGT extract, 200 mg/kg fGT extract, and 100 mg/kg fGT groups, respectively, compared with db control mice (Table 1). Daily water consumptions were changed by −21.77%, −19.09%, −40.86%, −28.23% and −23.92% at 83 days after initial administration in the 250 mg/kg metformin, 400 mg/kg GT extracts, 400 mg/kg, 200 mg/kg, and 100 mg/kg fGT groups, respectively, compared with the db control group.

**Table 1:** Food and water consumptions in intact normoglycemic and db/db mice.

Groups	Food Consumption (g/24 h/Mouse): Days after Initial Treatment			Water Consumption (mL/24 h/Mouse): Days after Initial Treatment		
	28	63	83	28	63	83
*Controls*						
Intact	2.79 ± 0.30	2.59 ± 0.37	2.59 ± 0.35	13.75 ± 1.58	14.88 ± 3.48	14.63 ± 3.07
db	4.60 ± 0.77 ^a^	4.20 ± 0.52 ^f^	4.12 ± 0.45 ^a^	34.13 ± 2.95 ^f^	44.13 ± 2.17 ^a^	46.50 ± 3.89 ^a^
*Reference*						
Metformin	3.58 ± 0.69 ^ac^	3.43 ± 0.13 ^f^^g^	3.20 ± 0.16 ^ac^	28.50 ± 3.12 ^fg^	35.13 ± 2.95 ^ac^	36.38 ± 3.46 ^ac^
GT 400 mg/kg	3.60 ± 0.39 ^ac^	3.61 ± 0.31 ^fh^	3.45 ± 0.41 ^ac^	36.13 ± 7.70 ^f^	34.75 ± 2.05 ^ac^	37.63 ± 2.77 ^ac^
*fGT treated*						
400 mg/kg	3.52 ± 0.60 ^bc^	2.48 ± 0.16 ^gi^	2.67 ± 0.32 ^cd^	29.25 ± 2.76 ^fgj^	26.50 ± 3.38 ^acd^	27.50 ± 3.30 ^acd^
200 mg/kg	3.65 ± 0.63 ^ac^	2.79 ± 0.44 ^gi^	2.81 ± 0.33 ^cd^	25.38 ± 2.83 ^fgj^	29.63 ± 3.16 ^acd^	33.38 ± 2.77 ^ace^
100 mg/kg	3.61 ± 0.50 ^ac^	3.35 ± 0.47 ^fg^	3.48 ± 0.28 ^ac^	28.63 ± 3.54 ^fh^	32.50 ± 3.25 ^ac^	35.38 ± 3.66 ^ac^

Values are expressed mean ± S.D. of eight mice. GT, Green tea aqueous lyophilized extracts; fGT, Aquilariae Lignum-fermented green tea aqueous lyophilized extracts; metformin was administrated at a dose level of 250 mg/kg; ^a^ *p <* 0.01 and ^b^ *p <* 0.05 as compared with intact control by LSD test; ^c^ *p <* 0.01 as compared with db control by LSD test; ^d^ *p <* 0.01 and ^e^ *p <* 0.05 as compared with GT 400 mg/kg by LSD test; ^f^ *p <* 0.01 as compared with intact control by MW test; ^g^ *p <* 0.01 and ^h^ *p <* 0.05 as compared with db control by MW test; ^i^ *p <* 0.01 and ^j^ *p <* 0.05 as compared with GT 400 mg/kg by MW test.

#### 3.1.3. Effects on the Fecal Excretion

We observed increases of fecal excretion in the db control group compared with the intact control group. Conversely, significant increases in fecal excretion were observed in all test substance administered mice compared with db control group (Table 1). Especially, 400 and 200 mg/kg fGT extracts treated db/db mice also showed significant increases of fecal excretion as compared with GT extract 400 mg/kg treated db/db mice, at 28, 63 and 83 days after initial administration, respectively. However, 100 mg/kg fGT extracts treated mice did not showed any significant changes on the daily fecal excretion as compared with those of 400 mg/kg GT extract treated mice at all three measuring times (Table 2).

**Table 2:** Urine volumes and fecal excretion in intact normoglycemic and db/db mice.

Groups	Urine Volume (mL/24 h/Mouse): Days after Initial Treatment			Fecal Excretion (g/24 h/Mouse): Days after Initial Treatment		
	28	63	83	28	63	83
*Controls*						
Intact	1.53 ± 0.47	1.55 ± 0.23	1.54 ± 0.32	1.06 ± 0.32	1.12 ± 0.19	1.14 ± 0.17
db	9.91 ± 1.96 ^d^	16.84 ± 2.25 ^a^	17.78 ± 2.29 ^d^	1.39 ± 0.22 ^d^	1.40 ± 0.21 ^b^	1.40 ± 0.17 ^a^
*Reference*						
Metformin	7.45 ± 1.85 ^df^	13.16 ± 1.70 ^ab^	13.69 ± 1.35 ^de^	1.75 ± 0.27 ^de^	1.77 ± 0.26 ^ab^	1.75 ± 0.25 ^ab^
GT 400 mg/kg	7.88 ± 1.32 ^d^	13.83 ± 2.01 ^ab^	14.39 ± 0.91 ^de^	1.69 ± 0.17 ^df^	1.71 ± 0.16 ^ab^	1.66 ± 0.17 ^ab^
*fGT treated*						
400 mg/kg	5.74 ± 1.21 ^deg^	7.39 ± 2.11 ^abc^	7.65 ± 2.38 ^deg^	2.04 ± 0.07 ^deg^	2.18 ± 0.22 ^abc^	2.13 ± 0.14 ^abc^
200 mg/kg	5.86 ± 0.99 ^deg^	8.80 ± 1.79 ^abc^	9.19 ± 2.17 ^deg^	1.97 ± 0.16 ^deh^	2.01 ± 0.18 ^abc^	1.93 ± 0.14 ^abc^
100 mg/kg	7.08 ± 2.10 ^df^	12.43 ± 2.96 ^ab^	14.68 ± 1.34 ^de^	1.75 ± 0.33 ^df^	1.75 ± 0.24 ^ab^	1.74 ± 0.25 ^ab^

Values are expressed mean ± S.D. of eight mice. GT, Green tea aqueous lyophilized extracts; fGT, Aquilariae Lignum-fermented green tea aqueous lyophilized extracts; metformin was administrated at a dose level of 250 mg/kg; ^a^ *p <* 0.01 as compared with intact control by LSD test; ^b^ *p <* 0.01 as compared with db control by LSD test; ^c^ *p <* 0.01 as compared with GT 400 mg/kg by LSD test; ^d^ *p <* 0.01 as compared with intact control by MW test; ^e^ *p <* 0.01; ^f^ *p <* 0.05 as compared with db control by MW test; ^g^ *p <* 0.01; ^h^ *p <* 0.05 as compared with GT 400 mg/kg by MW test.

#### 3.1.4. Effects on the Periovarian Fat Weight, Periovarian Adipocyte, and Periovarian Fat Adiponectin Contents

**Figure 3:**
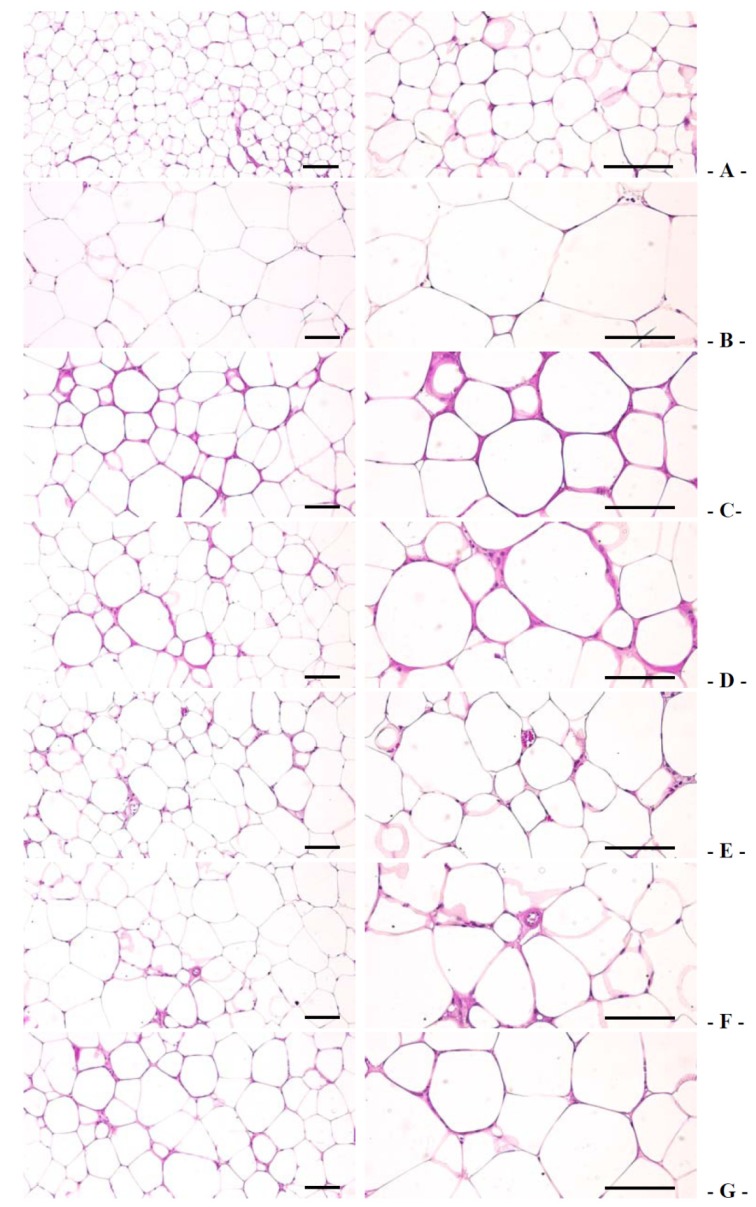
Representative histological images of the adipocytes, taken from intact normoglycemic or db/db mice periovarian fat pads. (**A**) Intact control mouse; (**B**) db control mouse; (**C**) metformin 250 mg/kg treated db mouse; (**D**) GT 400 mg/kg treated db mouse; (**E**) fGT 400 mg/kg treated db mouse; (**F**) fGT 200 mg/kg treated db mouse; (**G**) fGT 100 mg/kg treated db mouse. GT, Green tea aqueous lyophilized extracts; fGT, Aquilariae Lignum-fermented green tea aqueous lyophilized extracts. All Hematoxylin and Eosin stain. Scale bars = 80 µm.

Compared with the intact control group, the db control group showed an increase in the periovarian fat pad weight, periovarian white adipocyte diameters, and thicknesses of deposited fat pads, concomitantly with a decrease in fat tissue adiponectin levels. In notice, compared with that of the 400 mg/kg GT extract group, 400 and 200 mg/kg fGT treated mice more decreased in the periovarian fat pad weight, the periovarian white adipocyte diameters, and thicknesses of deposited fat pads, while periovarian fat tissue adiponectin contents more increased (Table 3, Table 4, Table 5 and Table 6, Figure 3). The relative periovarian fat pad weight in db control group was changed by 173.14% compared with the intact control group and was changed by −24.05%, −22.80%, −44.91%, −39.22% and −29.83% in 250 mg/kg metformin, 400 mg/kg GT extracts, 400 mg/kg, 200 mg/kg, and 100 mg/kg fGT groups, respectively, compared with the db control group. The deposited periovarian fat pad thicknesses in db control were changed as 774.34% as compared with intact control, but they were changed by −20.42%, −20.06%, −42.86%, −40.98% and −29.53% in 250 mg/kg metformin, 400 mg/kg GT extracts, 400, 200 and 100 mg/kg fGT treated db/db mice, respectively, compared with db control. The periovarian fat adiponectin contents in db control were changed as −66.88% as compared with intact control, but they were changed as 64.26%, 54.43%, 148.22%, 102.51% and 66.88% in 250 mg/kg metformin, 400 mg/kg GT extracts, 400, 200 and 100 mg/kg fGT treated db/db mice, respectively, compared with db control.

**Table 3:** Changes on absolute organ weight in intact normoglycemic and db/db mice.

Groups	Absolute Organ Weight (g)			
	Liver	Kidney	Periovarian Fats	Pancreas
*Controls*				
Intact	0.896 ± 0.020	0.114 ± 0.006	0.042 ± 0.019	0.103 ± 0.011
db	2.552 ± 0.121 ^f^	0.233 ± 0.017 ^f^	0.772 ± 0.097 ^a^	0.141 ± 0.017 ^a^
*Reference*				
Metformin	2.084 ± 0.118 ^fg^	0.207 ± 0.011 ^fg^	0.560 ± 0.082 ^ac^	0.131 ± 0.011 ^a^
GT 400 mg/kg	2.062 ± 0.107 ^fg^	0.208 ± 0.010 ^fg^	0.567 ± 0.102 ^ac^	0.135 ± 0.021 ^a^
*fGT treated*				
400 mg/kg	1.366 ± 0.352 ^fgh^	0.180 ± 0.005 ^fgh^	0.350 ± 0.067 ^ace^	0.123 ± 0.020 ^bd^
200 mg/kg	1.546 ± 0.257 ^fgh^	0.189 ± 0.014 ^fgh^	0.380 ± 0.058 ^ace^	0.127 ± 0.017 ^a^
100 mg/kg	1.949 ± 0.167 ^fg^	0.200 ± 0.016 ^fg^	0.486 ± 0.119 ^ac^	0.126 ± 0.018 ^a^

Values are expressed as mean ± S.D. of eight mice. GT, Green tea aqueous lyophilized extracts; fGT, Aquilariae Lignum-fermented green tea aqueous lyophilized extracts; metformin was administrated at a dose level of 250 mg/kg; ^a^ *p <* 0.01 and ^b^ *p <* 0.05 as compared with intact control by LSD test; ^c^ *p <* 0.01 and ^d^ *p <* 0.05 as compared with db control by LSD test; ^e^ *p <* 0.01 as compared with GT 400 mg/kg by LSD test; ^f^ *p <* 0.01 as compared with intact control by MW test; ^g^ *p <* 0.01 as compared with db control by MW test; ^h^ *p <* 0.01 as compared with GT 400 mg/kg by MW test.

**Table 4:** Changes on relative organ weight in intact normoglycemic and db/db mice.

Groups	Absolute Organ Weight (% of Body Weight)			
	Liver	Kidney	Periovarian Fats	Pancreas
*Controls*				
Intact	4.015 ± 0.198	0.512 ± 0.039	0.191 ± 0.089	0.459 ± 0.043
db	5.507 ± 0.202 ^d^	0.503 ± 0.029	1.667 ± 0.209 ^a^	0.305 ± 0.039 ^a^
*Reference*				
Metformin	4.940 ± 0.192 ^de^	0.490 ± 0.028	1.327 ± 0.185 ^ab^	0.311 ± 0.025 ^a^
GT 400 mg/kg	4.856 ± 0.280 ^de^	0.491 ± 0.033	1.333 ± 0.228 ^ab^	0.317 ± 0.045 ^a^
*fGT treated*				
400 mg/kg	3.715 ± 0.888 ^ef^	0.494 ± 0.042	0.953 ± 0.175 ^abc^	0.333 ± 0.037 ^a^
200 mg/kg	4.025 ± 0.731 ^eg^	0.491 ± 0.026	0.984 ± 0.132 ^abc^	0.329 ± 0.034 ^a^
100 mg/kg	4.691 ± 0.325 ^de^	0.483 ± 0.061	1.175 ± 0.308 ^ab^	0.303 ± 0.041 ^a^

Values are expressed as mean ± S.D. of eight mice. GT, Green tea aqueous lyophilized extracts; fGT, Aquilariae Lignum-fermented green tea aqueous lyophilized extracts; metformin was administrated at a dose level of 250 mg/kg; ^a^ *p <* 0.01 as compared with intact control by LSD test; ^b^ *p <* 0.01 as compared with db control by LSD test; ^c^ *p <* 0.01 as compared with GT 400 mg/kg by LSD test; ^d^ *p <* 0.01 as compared with intact control by MW test; ^e^ *p <* 0.01 as compared with db control by MW test; ^f^ *p <* 0.01 and ^g^ *p <* 0.05 as compared with GT 400 mg/kg by MW test.

**Table 5:** Serum leptin and adiponectin levels with periovarian fat adiponectin contents in intact normoglycemic and db/db mice.

Groups	Serum Levels		Fat Adiponectin Contents (Relative of db Control)
	Leptin (ng/mL)	Adiponectin (μg/mL)	
*Controls*			
Intact	2.80 ± 1.35	29.82 ± 5.10	305.65 ± 24.55
db	34.08 ± 10.16 ^d^	11.04 ± 2.57 ^a^	102.22 ± 5.19 ^d^
*Reference*			
Metformin	21.04 ± 3.18 ^df^	19.52 ± 3.68 ^ab^	166.27 ± 19.64 ^df^
GT 400 mg/kg	21.86 ± 3.56 ^df^	17.60 ± 3.38 ^ab^	156.31 ± 26.92 ^df^
*fGT treated*			
400 mg/kg	11.29 ± 3.57 ^dfg^	26.72 ± 4.61 ^bc^	251.25 ± 42.33 ^efg^
200 mg/kg	16.00 ± 3.00 ^dfg^	23.85 ± 4.45 ^abc^	204.98 ± 21.40 ^dfg^
100 mg/kg	20.40 ± 4.62 ^df^	18.64 ± 4.04 ^ab^	168.92 ± 29.20 ^df^

Values are expressed as mean ± S.D. of eight mice. GT, Green tea aqueous lyophilized extracts; fGT, Aquilariae Lignum-fermented green tea aqueous lyophilized extracts; metformin was administrated at a dose level of 250 mg/kg; ^a^ *p <* 0.01 as compared with intact control by LSD test; ^b^ *p <* 0.01 as compared with db control by LSD test; ^c^ *p <* 0.01 as compared with GT 400 mg/kg by LSD test; ^d^ *p <* 0.01 and ^e^ *p <* 0.05 as compared with intact control by MW test; ^f^ *p <* 0.01 as compared with db control by MW test; ^g^ *p <* 0.01 as compared with GT 400 mg/kg by MW test.

**Table 6:** Changes on histopathology-histomorphometry of the liver, kidney and periovarian fat pads in intact normoglycemic and db/db mice.

Groups	Liver Steatosis (%/mm^2^ of Hepatic Tissues)	Mean Hepatocyte Diameters (μm/Cell)	Degenerative Glomerulus Numbers (%)	Mean Thickness of Periovarian Fat Pads (mm)	Mean Diameters of Adipocyte (μm/Cell)
*Controls*					
Intact	7.95 ± 5.55	15.94 ± 3.40	4.50 ± 3.34	2.19 ± 0.58	57.90 ± 12.61
db	81.71 ± 6.95 ^a^	35.75 ± 4.01 ^e^	74.25 ± 11.02 ^a^	7.40 ± 0.74 ^a^	158.15 ± 13.05 ^a^
*Reference*					
Metformin	53.85 ± 10.81 ^ac^	22.14 ± 1.89 ^ef^	54.13 ± 10.66 ^ac^	4.98 ± 0.26 ^ac^	120.12 ± 17.31 ^ac^
GT 400 mg/kg	57.27 ± 8.01 ^ac^	25.12 ± 1.51 ^ef^	60.75 ± 11.76 ^ac^	5.70 ± 0.69 ^ac^	122.09 ± 25.59 ^ac^
*fGT treated*					
400 mg/kg	18.71 ± 6.16 ^acd^	17.96 ± 1.60 ^fg^	23.00 ± 4.72 ^acd^	2.95 ± 0.67 ^bcd^	87.12 ± 17.96 ^acd^
200 mg/kg	31.30 ± 5.87 ^acd^	19.09 ± 1.25 ^fg^	36.00 ± 5.71 ^acd^	4.40 ± 0.57 ^acd^	96.12 ± 9.98 ^acd^
100 mg/kg	53.78 ± 8.54 ^ac^	23.82 ± 3.47 ^ef^	55.38 ± 7.76 ^ac^	5.41 ± 0.90 ^ac^	110.97 ± 15.56 ^ac^

Values are expressed as mean ± SD of eight mice. GT, Green tea aqueous lyophilized extracts; fGT, Aquilariae Lignum-fermented green tea aqueous lyophilized extracts; metformin was administrated at a dose level of 250 mg/kg; ^a^ *p <* 0.01 and ^b^ *p <* 0.05 as compared with intact control by LSD test; ^c^ *p <* 0.01 as compared with db control by LSD test; ^d^ *p <* 0.01 as compared with GT 400 mg/kg by LSD test; ^e^ *p <* 0.01 as compared with intact control by MW test; ^f^ *p <* 0.01 as compared with db control by MW test; ^g^ *p <* 0.01 as compared with GT 400 mg/kg by MW test.

#### 3.1.5. Effects on the Serum Leptin Levels

The serum leptin levels were significantly increased in the db control group compared with the intact control group. The serum leptin levels of 400 and 200 mg/kg fGT extracts group more decreased compared with that of the 400 mg/kg GT extract group (Table 5). The leptin levels in db control were changed as 1116.06% as compared with intact control, while they were changed by −38.26%, −35.85%, −66.88%, −53.05% and −40.14% in 250 mg/kg metformin, 400 mg/kg GT extracts, 400 mg/kg, 200 mg/kg, and 100 mg/kg fGT groups, respectively, compared with the db control group.

#### 3.1.6. Effects on the Exocrine Pancreas Zymogen Granule Contents

**Figure 4:**
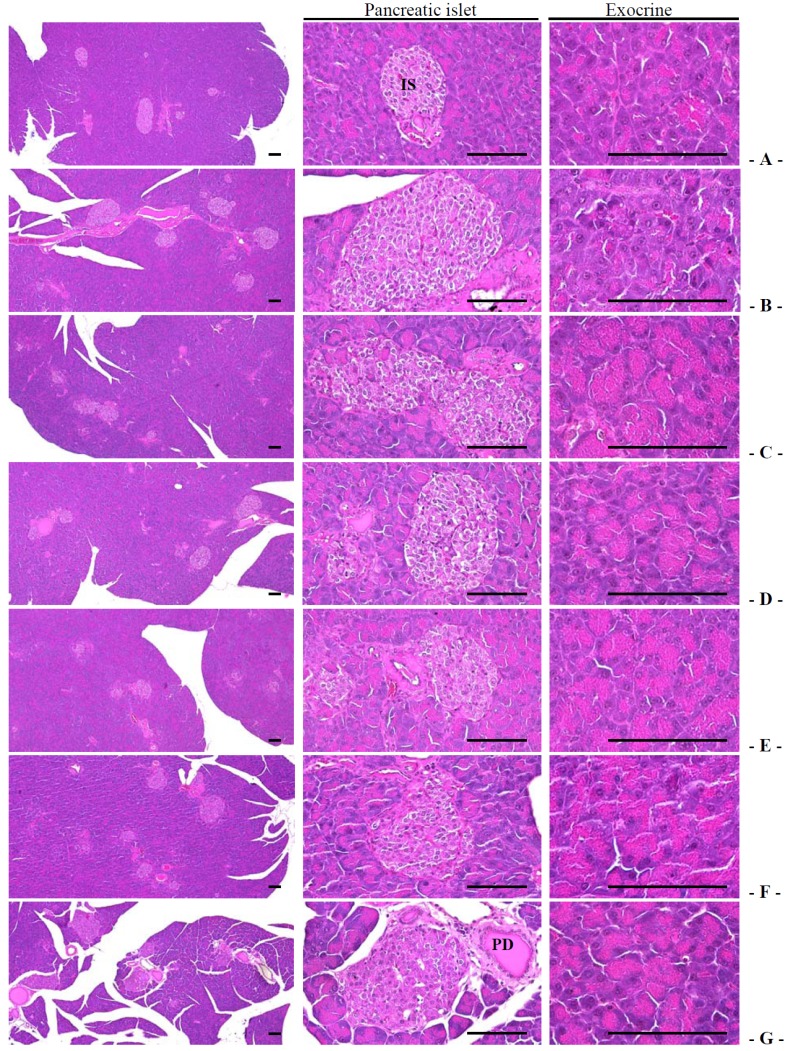
Representative histological images of the pancreas, taken from intact normoglycemic or db/db mice. (**A**) Intact control mouse; (**B**) db control mouse; (**C**) metformin 250 mg/kg treated db mouse; (**D**) GT 400 mg/kg treated db mouse; (**E**) fGT 400 mg/kg treated db mouse; (**F**) fGT 200 mg/kg treated db mouse; (**G**) fGT 100 mg/kg treated db mouse. GT, Green tea aqueous lyophilized extracts; fGT, Aquilariae Lignum-fermented green tea aqueous lyophilized extracts; IS, pancreatic islet; PD, pancreatic secretory duct. All Hematoxylin and Eosin stain. Scale bars = 80 µm.

Exocrine pancreatic zymogen granule content (percentage of the exocrine pancreas occupied by zymogen granules) was significantly decreased in db control mice compared with intact control mice. The treatment of 400 and 200 mg/kg fGT extract showed significant increases in the percentage of the exocrine pancreas occupied by zymogen granules compared with db/db mice treated with 400 mg/kg GT extract (Table 7, Figure 4). The percentage regions of exocrine pancreas occupied by zymogen granule in db control were changed as −43.80% as compared with intact control, but they were changed by 78.58%, 39.62%, 106.30%, 66.67% and 44.41% in 250 mg/kg metformin, 400 mg/kg GT extracts, 400, 200 and 100 mg/kg fGT treated db/db mice, respectively compared with db control.

**Table 7:** Changes on histopathology-histomorphometry of the pancreas in intact normoglycemic and db/db mice.

Groups	Zymogen Granules (%/mm^2^ of Exocrine)	Mean Islet Numbers (Numbers/10 mm^2^)	Mean Islet Diameter (μm/Islet)	Insulin-IR Cells (Cells/mm^2^ of Islet)	Glucagon-IR Cells (Cells/mm^2^ of Islet)	Insulin/Glucagon Ratio
*Controls*						
Intact	54.79 ± 7.40	10.38 ± 1.92	102.58 ± 20.29	502.88 ± 29.43	136.00 ± 10.16	3.71 ± 0.19
db	30.79 ± 7.88 ^a^	23.63 ± 2.50 ^a^	203.30 ± 19.26 ^a^	120.50 ± 15.66 ^f^	252.00 ± 23.32 ^a^	0.48 ± 0.08 ^f^
*Reference*						
Metformin	54.99 ± 9.60 ^c^	15.88 ± 2.17 ^ac^	146.70 ± 18.52 ^ac^	235.63 ± 54.83 ^fg^	191.13 ± 15.21 ^ac^	1.25 ± 0.35 ^fg^
GT 400 mg/kg	42.99 ± 3.01 ^ac^	16.75 ± 1.83 ^ac^	152.01 ± 11.75 ^ac^	224.38 ± 57.12 ^fg^	204.88 ± 22.22 ^ac^	1.11 ± 0.31 ^fg^
*fGT treated*						
400 mg/kg	63.52 ± 6.03 ^bcd^	11.38 ± 1.41 ^cd^	104.12 ± 16.16 ^cd^	378.88 ± 20.62 ^fgh^	153.25 ± 12.37 ^cd^	2.49 ± 0.25 ^fgh^
200 mg/kg	51.32 ± 4.21 ^ce^	14.13 ± 1.55 ^acd^	132.07 ± 11.56 ^ace^	328.38 ± 32.98 ^fgh^	169.75 ± 14.69 ^acd^	1.95 ± 0.29 ^fgh^
100 mg/kg	44.47 ± 6.42 ^ac^	16.38 ± 1.92 ^ac^	145.80 ± 22.01 ^ac^	239.75 ± 55.21 ^fg^	194.25 ± 23.77 ^ac^	1.26 ± 0.36 ^fg^

Values are expressed as mean ± SD of eight mice. GT, Green tea aqueous lyophilized extracts; fGT, Aquilariae Lignum-fermented green tea aqueous lyophilized extracts; metformin was administrated at a dose level of 250 mg/kg; IR, immunoreactive; ^a^ *p <* 0.01 and ^b^ *p <* 0.05 as compared with intact control by LSD test; ^c^ *p <* 0.01 as compared with db control by LSD test; ^d^ *p <* 0.01 and ^e^ *p <* 0.05 as compared with GT 400 mg/kg by LSD test; ^f^ *p <* 0.01 as compared with intact control by MW test; ^g^ *p <* 0.01 as compared with db control by MW test; ^h^ *p <* 0.01 as compared with GT 400 mg/kg by MW test.

### 3.2. Anti-Diabetic Hypoglycemic Effects

#### 3.2.1. Effects on the Blood Glucose Levels

Significant increases in blood glucose levels were detected in db control compared with intact control. However, the blood glucose levels were significantly reduced in all test mterials group compared with db control, respectively. Especially, 400 and 200 mg/kg fGT extracts treated db/db mice showed significant decreases of the blood glucose levels compared with 400 mg/kg GT extracts treated db/db mice, respectively (Table 8). The blood glucose levels in db control were changed as 410.26% as compared with intact control, but they were changed by −34.55%, −25.02%, −54.57%, −46.47% and −30.08% in 250 mg/kg metformin, 400 mg/kg GT extracts, 400, 200 and 100 mg/kg fGT treated db/db mice, respectively, compared with db control.

**Table 8:** Changes on serum biochemistry in intact normoglycemic and db/db mice.

Groups	Controls		Reference		fGT Treated (mg/kg)		
	Intact	db	Metformin	GT 400 mg/kg	400	200	100
AST (IU/L)	44.00 ± 19.26	453.00 ± 111.08 ^e^	283.00 ± 61.65 ^eg^	298.38 ± 66.02 ^eg^	160.75 ± 38.24 ^egi^	216.68 ± 39.96 ^egj^	271.50 ± 31.20 ^eg^
ALT (IU/L)	24.25 ± 6.18	191.13 ± 15.42 ^e^	150.13 ± 15.42 ^eg^	162.00 ± 13.86 ^eg^	83.13 ± 28.51 ^egi^	104.63 ± 17.86 ^egi^	153.25 ± 14.34 ^eg^
BUN (mg/dL)	29.88 ± 5.19	63.75 ± 16.21 ^e^	44.83 ± 5.85 ^eg^	47.38 ± 6.39 ^eh^	36.63 ± 4.93 ^fgi^	40.13 ± 2.17 ^egi^	44.25 ± 6.65 ^eg^
Creatinine (mg/dL)	0.64 ± 0.18	1.93 ± 0.36 ^a^	1.48 ± 0.21 ^ac^	1.49 ± 0.20 ^ac^	1.04 ± 0.21 ^acd^	1.14 ± 0.20 ^acd^	1.41 ± 0.18 ^ac^
Glucose (mg/dL)	120.63 ± 20.48	615.50 ± 86.67 ^a^	402.88 ± 62.99 ^ac^	461.50 ± 55.45 ^ac^	279.63 ± 104.50 ^acd^	329.50 ± 83.26 ^acd^	430.38 ± 64.38 ^ac^
TC (mg/dL)	120.50 ± 39.58	312.25 ± 38.34 ^e^	247.25 ± 11.99 ^eg^	266.00 ± 16.31 ^eg^	223.63 ± 16.54 ^egi^	236.88 ± 15.87 ^egi^	257.50 ± 20.47 ^eg^
TG (mg/dL)	85.25 ± 12.31	232.25 ± 23.32 ^a^	162.25 ± 18.58 ^ac^	175.38 ± 21.39 ^ac^	108.00 ± 16.20 ^bcd^	143.13 ± 21.24 ^acd^	165.00 ± 25.93 ^ac^
LDL (mg/dL)	11.50 ± 1.60	42.38 ± 5.45 ^e^	31.88 ± 5.64 ^eg^	32.38 ± 5.55 ^eg^	16.38 ± 4.14 ^fgi^	19.75 ± 2.12 ^egi^	28.75 ± 6.58 ^eg^
HDL (mg/dL)	68.13 ± 13.05	32.25 ± 10.15 ^e^	44.38 ± 5.01 ^eg^	43.00 ± 3.55 ^eg^	62.13 ± 8.32 ^gi^	56.88 ± 13.08 ^gj^	44.75 ± 5.85 ^eg^

Values are expressed as mean ± SD of eight mice. GT, Green tea aqueous lyophilized extracts; fGT, Aquilariae Lignum-fermented green tea aqueous lyophilized extracts; metformin was administrated at a dose level of 250 mg/kg; ALT, alanine aminotransferase; AST, aspartate aminotransferase; BUN, blood urea nitrogen; TC, total cholesterol; TG, triglyceride; LDL, low density lipoprotein; HDL, high density lipoprotein; ^a^ *p <* 0.01 and ^b^ *p <* 0.05 as compared with intact control by LSD test; ^e^ *p <* 0.01 and ^f^ *p <* 0.05 as compared with intact control by MW test; ^c^ *p <* 0.01 as compared with db control by LSD test; ^g^ *p <* 0.01 and ^h^ *p <* 0.05 as compared with db control by MW test; ^d^ *p <* 0.01 as compared with GT 400 mg/kg by LSD test; ^i^ *p <* 0.01 and ^j^ *p <* 0.05 as compared with GT 400 mg/kg by MW test.

#### 3.2.2. Effects on the Pancreatic Weight

Pancreas absolute weight was significantly increased in db control mice compared with intact control mice. Absolute pancreatic weight was decreased significantly in 400 mg/kg fGT treated mice compared with db control mice (Table 3 and Table 4). The relative pancreas weight in db control was changed as −33.69% as compared with intact control, but they were changed by 2.03%, 4.01%, 9.31%, 7.91% and −0.55% in 250 mg/kg metformin, 400 mg/kg GT extracts, 400, 200 and 100 mg/kg fGT treated db/db mice, respectively, compared with db control.

#### 3.2.3. Effects on the Pancreatic Islet Hyperplasia and Expansions

Significant increases of pancreatic islet numbers and mean diameters were observed in db control compared with intact control. These observations result from marked hyperplasia of pancreatic islet itself or component endocrine cells. The treatment of 400 and 200 mg/kg fGT extract showed significant decreases of the pancreatic islet numbers and mean diameters compared with 400 mg/kg GT extracts treated db/db mice (Table 7, Figure 4). The mean pancreatic islet numbers in db control were changed as 127.71% as compared with intact control, but they were changed by −32.80%, −29.10%, −51.85%, −40.21% and −30.69% in metformin 250 mg/kg, GT extracts 400 mg/kg, fGT 400, 200 and 100 mg/kg treated db/db mice as compared with db control, respectively. The percentages of islet occupied regions in db control were changed as 98.18% as compared with intact control, but they were changed by −27.84%, −25.23%, −48.79%, −35.03% and −28.28% in 250 mg/kg metformin, 400 mg/kg GT extracts, 400, 200 and 100 mg/kg fGT treated db/db mice, respectively, compared with db control.

#### 3.2.4. Effects on the Pancreatic Islet Insulin and Glucagon Cells

**Figure 5:**
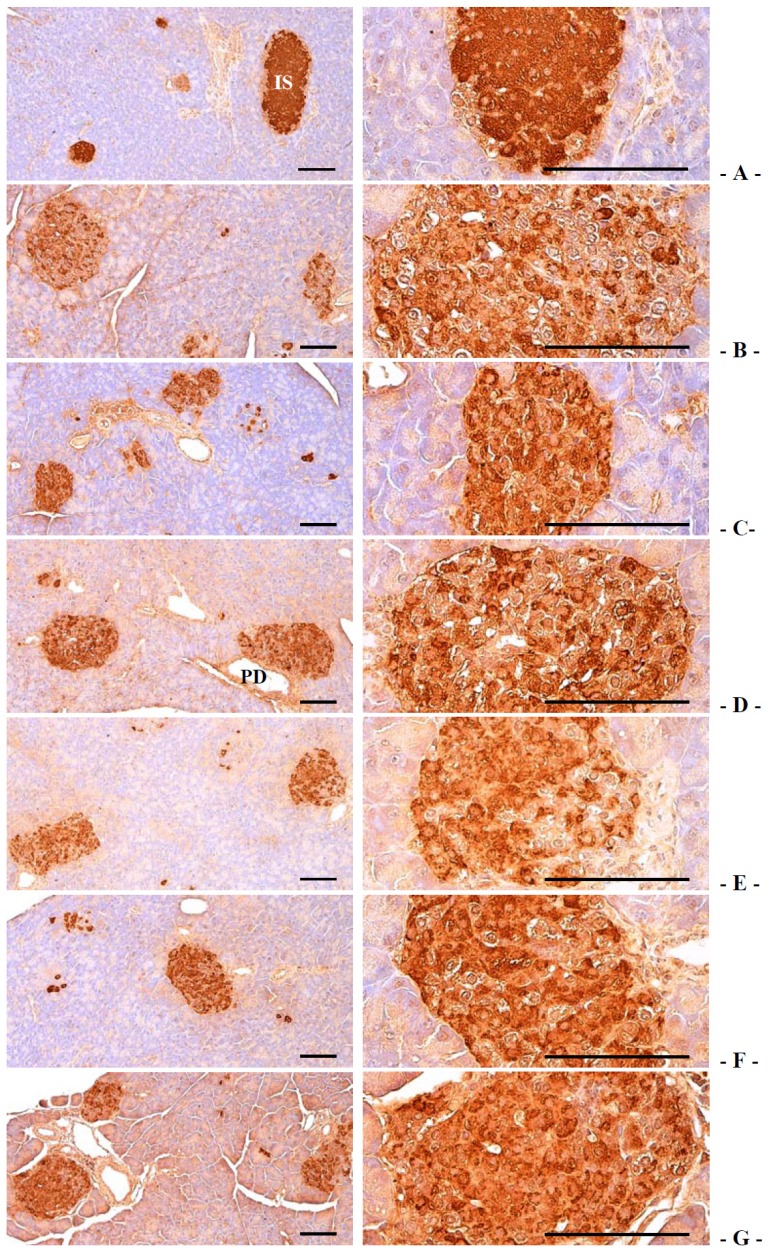
Representative histological images of the insulin-immunoreactive cells in the pancreas, taken from intact normoglycemic or db/db mice. (**A**) Intact control mouse; (**B**) db control mouse; (**C**) metformin 250 mg/kg treated db mouse; (**D**) GT 400 mg/kg treated db mouse; (**E**) fGT 400 mg/kg treated db mouse; (**F**) fGT 200 mg/kg treated db mouse; (**G**) fGT 100 mg/kg treated db mouse. GT, Green tea aqueous lyophilized extracts; fGT, Aquilariae Lignum-fermented green tea aqueous lyophilized extracts; IS, pancreatic islet; PD, pancreatic secretory duct. All immunostained by avidin-biotin-peroxidase complex. Scale bars = 80 µm.

**Figure 6:**
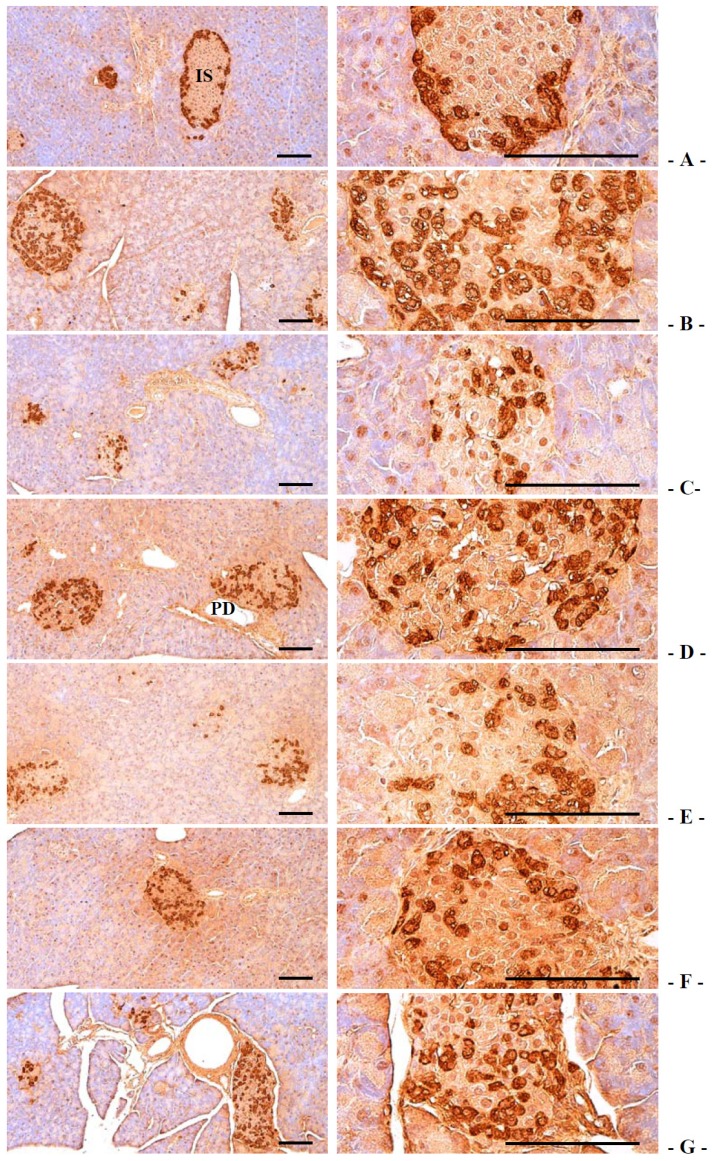
Representative histological images of the glucagon-immunoreactive cells in the pancreas, taken from intact normoglycemic or db/db mice. (**A**) Intact control mouse; (**B**) db control mouse; (**C**) metformin 250 mg/kg treated db mouse; (**D**) GT 400 mg/kg treated db mouse; (**E**) fGT 400 mg/kg treated db mouse; (**F**) fGT 200 mg/kg treated db mouse; (**G**) fGT 100 mg/kg treated db mouse. GT, Green tea aqueous lyophilized extracts; fGT, Aquilariae Lignum-fermented green tea aqueous lyophilized extracts; IS, pancreatic islet; PD, pancreatic secretory duct. All immunostained by avidin-biotin-peroxidase complex. Scale bars = 80 µm.

Compared with intact control, insulin-immunoreactive cells were significantly decreased in db control mice, while glucagon-immunoreactive cells were increased in db control. In addition, insulin/glucagon cells were significantly decreased. In notice, compared with 400 mg/kg GT extracts treated db/db mice, 400 and 200 mg/kg fGT extracts treated db/db mice showed significant increases of the insulin-immunolabelled cells and normalized insulin/glucagon cells and decreases of glucagon-positive cells (Table 7, Figure 5 and Figure 6). The mean numbers of insulin-immunoreactive cells in db control were changed as −76.04% as compared with intact control, but they were changed by 95.54%, 86.20%, 214.42%, 172.51% and 98.96% in 250 mg/kg metformin, 400 mg/kg GT extracts, 400, 200 and 100 mg/kg fGT treated db/db mice compared with db control, respectively. The mean numbers of glucagon-immunoreactive cells in db control were changed as 85.29% as compared with intact control, but they were changed by −24.16%, −18.70%, −39.19%, −32.64% and −22.92% in metformin 250 mg/kg, GT extracts 400 mg/kg, fGT 400, 200 and 100 mg/kg treated db/db mice, respectively, compared with db control. The insulin/glucagon cells in db control were changed as −86.99% as compared with intact control, but they were changed by 159.13%, 130.43%, 416.17%, 304.78% and 161.46% in 250 mg/kg metformin, 400 mg/kg GT extracts, 400, 200 and 100 mg/kg fGT treated db/db mice, respectively, as compared with db control.

### 3.3. Effects on Hyperlipidemia

#### 3.3.1. Effects on the Serum TC and TG Levels

Serum TC and TG levels were significantly increased in db control compared with intact control. However, the serum TC and TG levels were significantly decreased in all test substances group compared with db control group. Especially, 400 and 200 mg/kg fGT extracts treated db/db mice showed significant decreases in the serum TC and TG levels compared with 400 mg/kg GT extracts treated db/db mice (Table 8). The serum TC levels in db control were changed as 159.13% as compared with intact control, but they were changed by −20.82%, −14.81%, −28.38%, −24.14% and −17.53% in 250 mg/kg metformin, 400 mg/kg GT extracts, 400, 200 and 100 mg/kg fGT treated db/db mice, respectively, compared with db control. The serum triglyceride levels in db control were changed as 172.43% as compared with intact control, but they were changed by −30.14%, −24.49%, −53.50%, −38.37% and −28.96% in 250 mg/kg metformin, 400 mg/kg GT extracts, 400, 200 and 100 mg/kg fGT treated db/db mice, respectively, compared with db control.

#### 3.3.2. Effects on the Serum LDL and HDL Levels

Serum LDL levels were significantly increased, while serum HDL levels were significantly decreased in db control compared with intact control. However, the serum LDL levels were significantly decreased in fGT 400 mg/kg treated mice compared with db control, respectively. The treatment of 400 and 200 mg/kg fGT extract also showed significant decreases of the serum LDL levels while the serum HDL levels significantly increased compared with GT extracts 400 mg/kg treated db/db mice, respectively (Table 8). The serum LDL levels in db control were changed as 268.48% as compared with intact control, but they were changed by −24.78%, −23.60%, −61.36%, −53.39% and −32.15% in 250 mg/kg metformin, 400 mg/kg GT extracts, 400, 200 and 100 mg/kg fGT treated db/db mice, respectively, compared with db control. The serum HDL levels in db control were changed as −51.23% as compared with intact control, but they were changed by 37.60%, 33.33%, 92.64%, 76.36% and 38.76% in 250 mg/kg metformin, 400 mg/kg GT extracts, 400, 200 and 100 mg/kg fGT treated db/db mice, respectively, compared with db control.

#### 3.3.3. Effects on the Fecal TC and TG Levels

**Figure 7:**
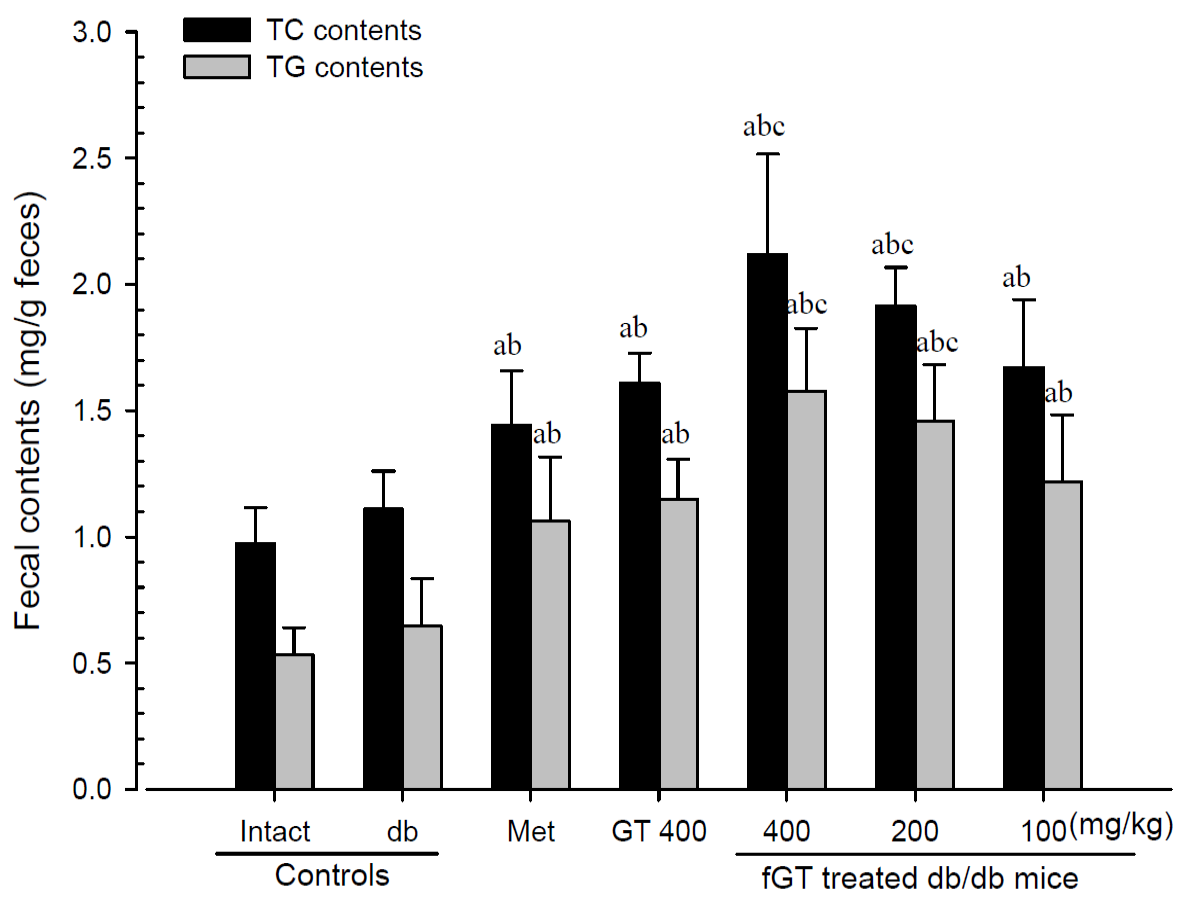
Fecal TC and TG content changes in intact normoglycemic and db/db mice. Values are expressed mean ± S.D. of eight mice. GT, Green tea aqueous lyophilized extracts; fGT, Aquilariae Lignum-fermented green tea aqueous lyophilized extracts; Met, metformin 250 mg/kg treated db/db mice; TC, total cholesterol; TG, triglyceride. ^a^ *p <* 0.01 as compared with intact control by LSD test; ^b^ *p <* 0.01 as compared with db control by LSD test; ^c^ *p <* 0.01 as compared with GT 400 mg/kg by LSD test.

Although slight increases of fecal TC and TG contents were detected in db control compared with intact control, the fecal TC and TG contents in all five test material treated mice were significantly elevated compared with db control mice. Especially, 400 and 200 mg/kg fGT extracts treated db/db mice also showed significant increases of the fecal TC and TG contents compared with 400 mg/kg GT extracts treated db/db mice (Figure 7). The fecal TC levels in db control were changed as 13.99% as compared with intact control, but they were changed by 29.95%, 44.93%, 90.88%, 72.41% and 50.56% in 250 mg/kg metformin, 400 mg/kg, GT extracts 400, 200 and 100 mg/kg fGT treated db/db mice, respectively, compared with db control. The fecal TG levels in db control were changed as 20.79% as compared with intact control, but they were changed by 64.41%, 77.95%, 143.71%, 125.73% and 88.39% in 250 mg/kg metformin, 400 mg/kg GT extracts, 400, 200 and 100 mg/kg fGT treated db/db mice, respectively, as compared with db control.

### 3.4. Effects on Hepatopathy

#### 3.4.1. Effects on the Liver Weight

Significant increases of liver absolute and relative weight were observed in db control compared with intact control. The treatment of 400 and 200 mg/kg fGT extract also showed significant decreases of the liver absolute and relative weight compared with 400 mg/kg GT extracts treated db/db mice (Table 3 and Table 4). The absolute liver weight in db control was changed as 184.83% as compared with intact control, but they were changed by −18.32%, −19.19%, −46.48%, −39.43% and −23.61% in 250 mg/kg metformin, 400 mg/kg GT extracts, 400, 200 and 100 mg/kg fGT treated db/db mice, respectively, compared with db control. The relative liver weight in db control was changed as 37.17% as compared with intact control, but they were changed by −10.29%, −11.82%, −32.55%, −26.92% and −14.82% in 250 mg/kg metformin, 400 mg/kg GT extracts, 400, 200 and 100 mg/kg fGT treated db/db mice, respectively, compared with db control.

#### 3.4.2. Effects on the Serum AST and ALT Levels

Compared with intact control, serum AST and ALT levels were significantly increased in db control. The treatment of 400 and 200 mg/kg fGT extract showed significant decreases of the serum AST levels compared with 400 mg/kg GT extracts treated db/db mice, respectively (Table 8). The serum AST levels in db control were changed as 929.55% as compared with intact control, but they were changed by −37.53%, −34.13%, −64.51%, −52.12% and −40.07% in 250 mg/kg metformin, 400 mg/kg GT extracts, 400, 200 and 100 mg/kg fGT treated db/db mice, respectively, compared with db control. The serum ALT levels in db control were changed as 688.14% as compared with intact control, but they were changed by −21.45%, −15.24%, −56.51%, −45.26% and −19.82% in 250 mg/kg metformin, 400 mg/kg GT extracts, 400, 200 and 100 mg/kg fGT treated db/db mice, respectively, compared with db control.

#### 3.4.3. Effects on the Steatohepatitis

**Figure 8:**
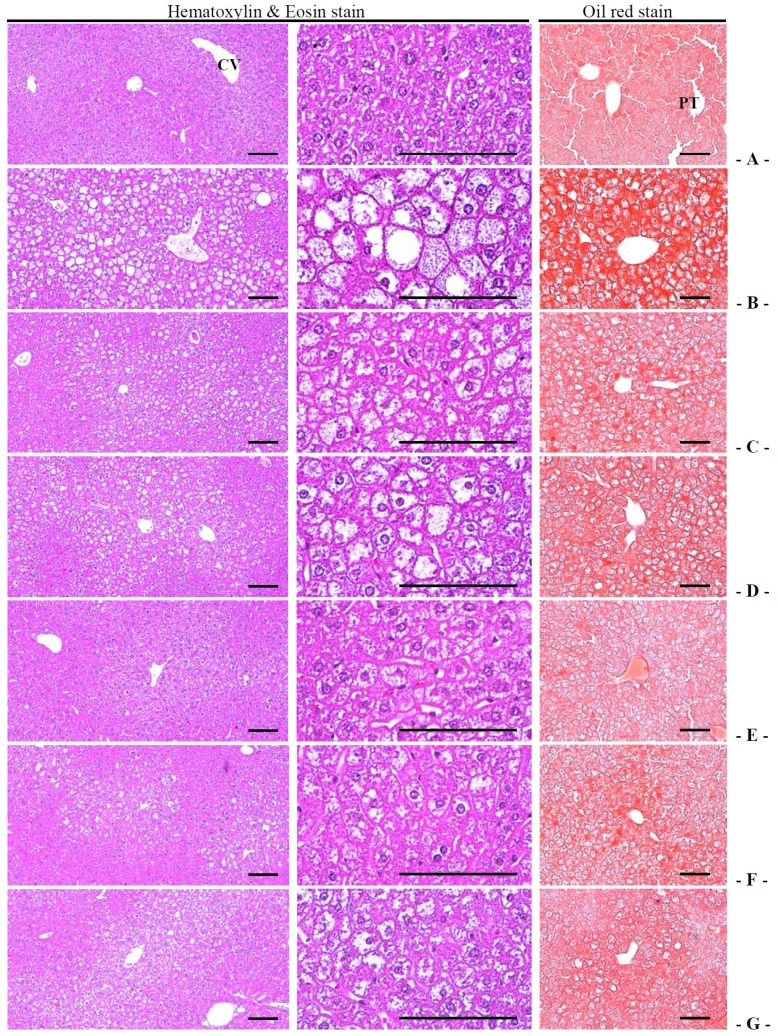
Representative histological images of the liver, taken from intact normoglycemic or db/db mice. (**A**) Intact control mouse; (**B**) db control mouse; (**C**) metformin 250 mg/kg treated db mouse; (**D**) GT 400 mg/kg treated db mouse; (**E**) fGT 400 mg/kg treated db mouse; (**F**) fGT 200 mg/kg treated db mouse; (**G**) fGT 100 mg/kg treated db mouse. GT, Green tea aqueous lyophilized extracts; fGT, Aquilariae Lignum-fermented green tea aqueous lyophilized extracts; CV, central vein; PT, portal triad. Scale bars = 80 µm. Hematoxylin and Eosin stain and Oil red stain.

Significant increases of steatohepatitis (percentages of fatty changed regions in liver parenchyma) were detected in db control compared with intact control, which result from severe hypertrophy of hepatocyte related to intracellular lipid depositions. The treatment of 400 and 200 mg/kg fGT extract also showed significant decreases of the steatohepatitis regions compared with 400 mg/kg GT extracts treated db/db mice (Table 6, Figure 8). The steatohepatitis regions in db control were changed as 923.55% as compared with intact control, but they were changed by −33.66%, −29.45%, −76.95%, −61.44% and −33.74% in 250 mg/kg metformin, 400 mg/kg GT extracts, 400, 200 and 100 mg/kg fGT treated db/db mice, respectively, compared with db control.

#### 3.4.4. Effects on the Hepatocyte Hypertrophy

Significant increases of mean hepatocyte diameters (hypertrophy) were detected in db control compared with intact control. The treatment of 400 and 200 mg/kg fGT extract also showed significant decreases of the hepatocyte hypertrophies compared with 400 mg/kg GT extracts treated db/db mice (Table 6, Figure 8). The mean hepatocyte diameters in db control were changed as 124.24% as compared with intact control, but they were changed by −38.08%, −29.72%, −49.75%, −46.61% and −33.38% in metformin 250 mg/kg, GT extracts 400 mg/kg, fGT 400, 200 and 100 mg/kg treated db/db mice, respectively, as compared with db control.

### 3.5. Effects on Nephropathy

#### 3.5.1. Effects on the Kidney Weight

Kidney absolute weight was significantly increased in db control compared with intact control, but they were significantly normalized by all five test materials compared with db/db mice. Especially, 400 and 200 mg/kg fGT extracts treated db/db mice also showed significant decreases of the kidney absolute weight compared with 400 mg/kg GT extracts treated db control mice, respectively (Table 3). While there is no meaningful changes on the kidney relative weight was demonstrated in all db/db mice as compared with intact normoglycemic control mice in the present study (Table 4).

#### 3.5.2. Effects on the Serum BUN and Creatinine Levels

Compared with intact control, serum BUN and creatinine levels were significantly increased in db control. The treatment of 400 and 200 mg/kg fGT extract showed significant decreases of the serum BUN and creatinine levels compared with 400 mg/kg GT extracts treated db/db mice, respectively (Table 8). The serum BUN levels in db control were changed as 113.39% as compared with intact control, but they were changed by −30.39%, −25.69%, −42.55%, −37.06% and −30.59% in 250 mg/kg metformin, 400 mg/kg GT extracts, 400, 200 and 100 mg/kg fGT treated db/db mice, respectively, as compared with db control. The serum creatinine levels in db control were changed as 201.96% as compared with intact control, but they were changed as −23.38%, −22.73%, −46.10%, −40.91% and −26.62% in metformin 250 mg/kg, GT extracts 400 mg/kg, fGT 400, 200 and 100 mg/kg treated db/db mice as compared with db control, respectively.

#### 3.5.3. Effects on the Urine Volume

The db control mice showed significant increases in mean daily urine excretion compared with intact control mice, at all three measured times, 28, 63 and 83 days after initial administration, respectively. The treatment of 400 and 200 mg/kg fGT extract showed significant decreases of urine excretion compared with 400 mg/kg GT extract treated db/db mice at 28, 63 and 83 days after initial administration, respectively (Table 2). The daily urine volumes in db control were changed as 550.00%, 986.29% and 1056.10% as compared with sham control at 28, 63 and 83 days after initial administration, and they were changed by −24.84%, −20.55%, −42.12%, −40.86% and −28.63% (at 28 days after initial administration), −21.83%, −17.89%, −56.12%, −47.74% and −26.21% (at 63 days after initial administration), and −23.00%, −19.06%, −56.96%, −48.31% and −17.44% (at 83 days after initial administration) in 250 mg/kg metformin, 400 mg/kg GT extracts, 400, 200 and 100 mg/kg fGT treated db/db mice, respectively, compared with db control.

#### 3.5.4. Effects on the Kidney Histopathology

**Figure 9:**
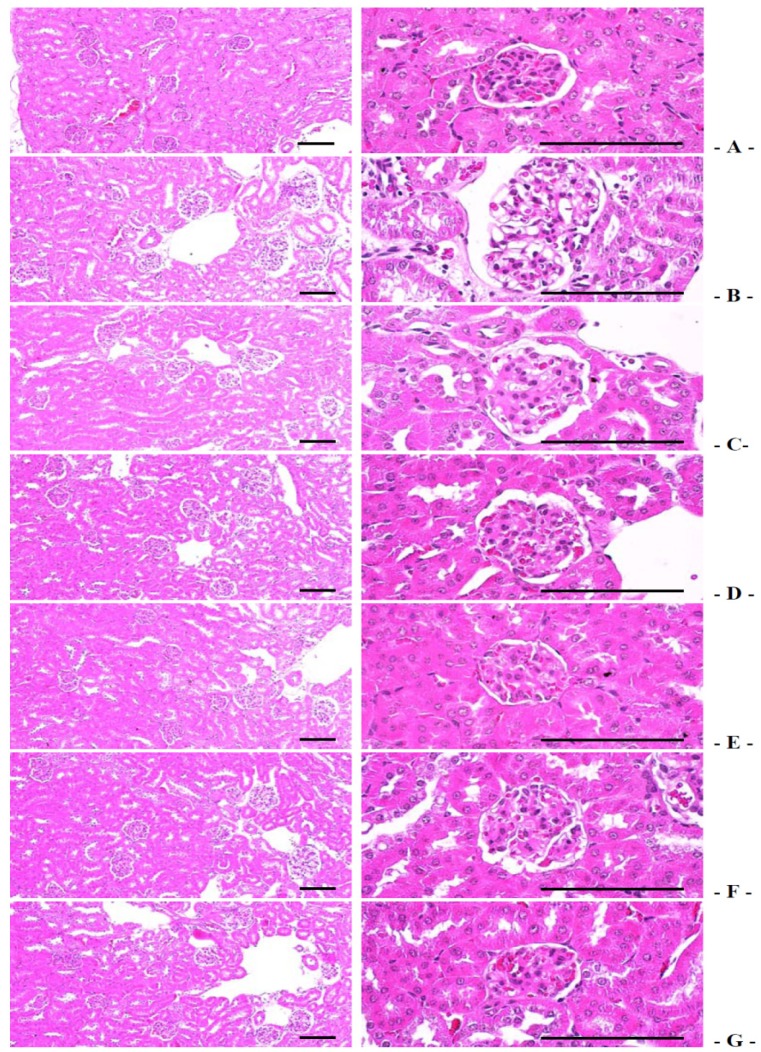
Representative histological images of the kidney, taken from intact normoglycemic or db/db mice. (**A**) Intact control mouse; (**B**) db control mouse; (**C**) metformin 250 mg/kg treated db mouse; (**D**) GT 400 mg/kg treated db mouse; (**E**) fGT 400 mg/kg treated db mouse; (**F**) fGT 200 mg/kg treated db mouse; (**G**) fGT 100 mg/kg treated db mouse. GT, Green tea aqueous lyophilized extracts; fGT, Aquilariae Lignum-fermented green tea aqueous lyophilized extracts. All Hematoxylin and Eosin stain. Scale bars = 80 µm.

The degenerative vasodilated atrophic glomerulus significantly increased in db control compared with intact control, which result from diabetic nephropathies. The treatment of 400 and 200 mg/kg fGT extract also showed significant decreases of the vasodilated atrophic glomerulus numbers compared with 400 mg/kg GT extracts treated db/db mice, respectively (Table 6, Figure 9). The degenerative glomerulus numbers in db control were changed as 1550.00% as compared with intact control, but they were changed by −27.10%, −18.18%, −69.02%, −51.52% and −25.42% in 250 mg/kg metformin, 400 mg/kg GT extracts, 400, 200 and 100 mg/kg fGT treated db/db mice, respectively, compared with db control.

### 3.6. Effects on Liver Lipid Peroxidation and Antioxidant Defense System

#### 3.6.1. Effects on the Liver Lipid Peroxidation

The liver lipid peroxidation and the hepatic MDA content elevations significantly increased in db control compared with intact control, but they were significantly normalized by all five test materials treated mice compared with db control mice, respectively. Especially, 400 and 200 mg/kg fGT extracts treated db/db mice also showed significant decreases of the hepatic lipid peroxidation compared with 400 mg/kg GT extracts treated db/db mice, respectively (Table 9). The hepatic lipid peroxidation in db control were changed as 278.26% as compared with intact control, but they were changed by −33.84%, −30.98%, −59.92%, −49.39% and −34.89% in 250 mg/kg metformin, 400 mg/kg GT extracts, 400, 200 and 100 mg/kg fGT treated db/db mice, respectively, compared with db control.

**Table 9:** Changes on the liver lipid peroxidation and antioxidant defense systems in intact normo-glycemic and db/db mice.

Groups	Lipid Peroxidation	Antioxidant Defense System		
	Malondialdehyde (nM/mg Tissue)	Glutathion (μM/mg Tissue)	Catalase (U/mg Tissue)	SOD (U/mg Tissue)
*Controls*				
Intact	17.47 ± 2.32	28.24 ± 4.76	17.46 ± 3.34	2.13 ± 0.29
db	66.09 ± 15.87 ^d^	10.31 ± 2.27 ^d^	8.17 ± 1.46 ^d^	0.71 ± 0.24 ^a^
*Reference*				
Metformin	43.73 ± 5.09 ^df^	14.10 ± 2.48 ^df^	10.82 ± 1.21 ^df^	1.04 ± 0.12 ^ab^
GT 400 mg/kg	45.62 ± 8.21 ^df^	15.55 ± 2.31 ^df^	11.83 ± 1.72 ^df^	1.11 ± 0.13 ^ab^
*fGT treated*				
400 mg/kg	26.49 ± 7.82 ^dfh^	24.52 ± 5.05 ^fh^	15.99 ± 1.72 ^fh^	1.55 ± 0.18 ^abc^
200 mg/kg	33.45 ± 6.97 ^dfi^	19.37 ± 1.35 ^dfh^	15.39 ± 1.91 ^fh^	1.43 ± 0.14 ^abc^
100 mg/kg	43.03 ± 12.77 ^dg^	16.23 ± 2.99 ^df^	12.44 ± 3.77 ^eg^	1.16 ± 0.25 ^ab^

Values are expressed as mean ± SD of eight mice. GT, Green tea aqueous lyophilized extracts; fGT, Aquilariae Lignum-fermented green tea aqueous lyophilized extracts; metformin was administrated at a dose level of 250 mg/kg; SOD, superoxide dismutase; ^a^ *p <* 0.01 as compared with intact control by LSD test; ^b^ *p <* 0.01 as compared with db control by LSD test; ^c^ *p <* 0.01 as compared with GT 400 mg/kg by LSD test; ^d^ *p <* 0.01 and ^e^ *p <* 0.05 as compared with intact control by MW test; ^f^ *p <* 0.01 and ^g^ *p <* 0.05 as compared with db control by MW test; ^h^ *p <* 0.01 and ^i^ *p <* 0.05 as compared with GT 400 mg/kg by MW test.

#### 3.6.2. Effects on the Hepatic GSH Contents

Significant decreases of hepatic GSH, a representative endogenous antioxidant, contents were detected in db control compared with intact control. The treatment of 400 and 200 mg/kg fGT extract also showed significant increases of the hepatic GSH contents compared with 400 mg/kg GT extracts treated db/db mice, respectively (Table 9). The hepatic GSH contents in db control were changed as −63.48% as compared with intact control, but they were changed as 36.77%, 50.77%, 137.77%, 87.82% and 57.41% in 250 mg/kg metformin, 400 mg/kg GT extracts, 400, 200 and 100 mg/kg fGT treated db/db mice, respectively, as compared with db control.

#### 3.6.3. Effects on the Hepatic CAT and SOD Activity

Compared with intact control, hepatic CAT and SOD, representative endogenous antioxidant enzymes, activities significantly decreased in db control. The treatment of 400 and 200 mg/kg fGT extract also showed significant increases of the hepatic CAT and SOD activities compared with 400 mg/kg GT extracts treated db/db mice, respectively (Table 9). The hepatic CAT activities in db control were changed as −53.20% as compared with intact control, but they were changed by 32.35%, 44.72%, 95.59%, 88.35% and 52.22% in 250 mg/kg metformin, 400 mg/kg GT extracts, 400, 200 and 100 mg/kg fGT treated db/db mice, respectively, as compared with db control. The hepatic SOD activities in db control were changed as −66.65% as compared with intact control, but they were changed by 46.05%, 55.54%, 118.28%, 100.88% and 63.09% in 250 mg/kg metformin, 400 mg/kg GT extracts, 400, 200 and 100 mg/kg fGT treated db/db mice, respectively, as compared with db control.

## 4. Discussion

Type 2 diabetes is a metabolic disorder with various pathological manifestations and is closely associated with abnormal glucose and lipid metabolism. Previous studies reported that GT can reduce blood glucose levels in diabetic db/db mice and glucose metabolism in healthy humans. An aqueous solution of GT polyphenols was also found to exert an anti-diabetic effect by reducing oxidative stress [38]. It has been shown that the major components of tea may change significantly according to the fermentation process, and consequently the effects of different teas vary [23]. We examined whether fermented GT with Aquilariae Lignum shows a stronger anti-diabetic effect than does unfermented GT on obese db/db mice with type 2 diabetes.

First, we examined body mass and food and water consumption in db/db mice. Control mice showed noticeable increases in body weight and food and water consumption. These changes were inhibited by fGT and GT extracts and by metformin. Furthermore, 400 and 200 mg/kg fGT extract showed stronger anti-obesity effects than did 400 mg/kg GT extract. It is assumed that these effects are related to increases in the modulatory effects on leptin receptor sensitivity. db/db Mice, homozygous for a point mutation in the leptin receptor gene, are a wildly used genetic model of type 2 diabetes, since they exhibit most of the characteristics of human type 2 diabetes patients, including hyperglycemia, dyslipidemia, and insulin resistance [39]. Since leptin receptor sensitivity and food and water consumption were normalized, and fat accumulation and hypertrophic changes in adipocytes were consequently decreased, the voracity of these obese mice with type 2 diabetes may have been inhibited.

Based on the above results, to verify the synergistic anti-obesity effect, we examined the effects of fGT extract on adipose tissues, adipocytes, serum leptin levels, and serum and periovarian fat adiponectin contents in db/db mice. Our results indicate that 400 and 200 mg/kg fGT extract had enhanced anti-obesity effects on db/db mice (decreased adipose tissue accumulation and adipocyte hypertrophy, decreased serum leptin levels, and increased serum and periovarian fat adiponectin contents) compared with GT extract. In general, an increase in adipose tissue accumulation is a feature of obesity development. In obesity, adipocytes show hypertrophic features in histopathological examinations [40]. Adiopose tissue produces diverse adipokines and releases them into the systemic circulation. The adipokine leptin is predominantly produced by adipose tissue. Leptin binds to the long-form of the leptin receptor (Ob-Rb) in the hypothalamus to reduce neuropeptide-Y and agouti-regulated protein (AgRP) activity and to increase pro-opiomelanocortin and cocaine- and amphetamine-related protein neuronal activity, thereby effectively decreasing appetite and food intake [41]. Adiponectin, a novel adipokine [42], is expressed exclusively in adipose tissue [42]. Recent studies found that hypoadiponectinemia is closely related to insulin resistance [43]. Moreover, it has been reported that obesity decreases plasma adiponectin levels in humans [44] and experimental animals [45]. The anti-diabetic drug thiazolidinedione stimulates endogenous adiponectin production in rodents and humans [46]. This evidence supports our observations that fGT-induced adipokines might be effective for preventing obesity and insulin resistance.

Obesity results in pancreatic steatosis, acinar cell atrophy, and a reduction in the number of zymogen granules [47]. The increased zymogen granule numbers in exocrine pancreatic acinar cells result in increased production of digestive enzymes for the digestion of lipids and proteins [48]. We found that 400 and 200 mg/kg fGT extract showed stronger inhibitory effects on pancreatic digestive enzyme release and a diminishment in zymogen deposition in the exocrine pancreas, compared with 400 mg/kg GT extract. Furthermore, fecal excretion and lipid contents (TC and TG) were more strongly inhibited by fGT extract than by GT extract. We presume that the inhibition of lipid digestion may be mediated by decreased pancreatic enzyme production or release. Further studies should aim to elucidate the detailed mechanisms behind the effects of fGT to exclude the possibility that fGT extract increases digestive tract motility. This effect is explained on the basis that an increase in digestive tract motility facilitates fecal excretion and consequently decreases body weight [49].

Hyperglycemia is the main sign of diabetes, and db/db mice exhibit hyperglycemia. During the progression of type 2 diabetes [50], hypertrophy or hyperplasia of endocrine pancreatic cells is related to insulin resistance. In db/db mice, the ratio of insulin- to glucagon-producing cells in the endocrine pancreas is decreased due to the destruction of insulin-producing β-cells and increases in glucagon-producing A cells [51]. In this study, 400 and 200 mg/kg fGT extract more strongly inhibited the histopathological alterations characterizing endocrine pancreatic changes, compared with 400 mg/kg GT extract. fGT extract markedly decreased pancreatic islet increases and expansion and increased the ratio of insulin- to glucagon-producing cells by inhibiting insulin-producing endocrine pancreatic cells and normalizing glucagon cell proliferation, as shown by histopathological analysis. These results indicate that fGT extract can effectively regulate hyperglycemia, which should be controlled in diabetes.

Since the most critical issues associated with hyperlipidemia are increased serum LDL, TG, and TC levels and decreased HDL levels [52], the efficacy of hypolipidemic agents is generally evaluated based on the decreases in serum LDL, TG, and TC levels and increase in HDL level [52]. Compared with control mice, fGT-treated mice showed decreased serum LDL, TG, and TC levels, and increased serum HDL levels. Notably, 400 and 200 mg/kg fGT extract showed more favorable inhibitory effects on hyperlipidemia compared with 400 mg/kg GT extract. This suggests that fermentation with Aquilariae Lignum synergistically increased the hypolipidemic effects of GT in db/db mice. These effects may be mediated by inhibition of lipid digestion as a result of decreased pancreatic enzyme production or release. The hypolipidemic effects of test substances in these db/db mice result from decreased lipid absorption and propulsion into the feces through pancreatic digestive enzyme-modulating effects (as mentioned above).

Diabetic nephropathy is a serious complication of diabetes mellitus, which is the leading cause of end-stage renal disease. In chronic diabetes, kidney weight is increased and serum blood urea nitrogen (BUN) and creatinine levels are elevated due to swelling, inflammation, and necrotic processes. BUN testing measures the amount of urea nitrogen in the blood. Creatinine is a non-protein nitrogenous product of muscle metabolism. As with BUN, serum creatinine levels are elevated by conditions that reduce glomerular filtration [53]. Diabetic hepatopathy involves damage to the liver caused by diabetes. As diabetes progresses, the weight of the liver increases due to fibrosis or abnormal glycosylation related to hepatic steatosis. In addition, hypertrophic changes in the cytoplasm of hepatocytes due to lipid deposition were observed with the elevation of serum AST and ALT levels. Serum AST activity is elevated with skeletal muscle necrosis and hepatocellular necrosis. Elevated serum AST activity with no ALT elevation is an indicator of muscle necrosis. AST activity increases more slowly than does ALT activity in liver damage and indicates more complete cellular disruption, because it leaks only from cells with necrosis but not membrane instability [53]. ALT is present in large quantities in the cytoplasm of hepatocytes. This enzyme enters the blood when liver cells are damaged or destroyed and circulates for a few days. In this study, compared with 400 mg/kg GT extract, 400 and 200 mg/kg fGT extract effectively ameliorated diabetic nephropathy (kidney weight, BUN and creatine levels) and hepatopathy (liver weight, AST and ALT levels). This inhibition of abnormal alterations in the kidneys and liver suggests that fGT extract improved diabetic nephropathy and hepatopathy.

It is well established that free radicals contribute to the etiology of diabetes and alter antioxidant defense [54]. Hyperglycemia-generated free radicals are formed by glucose auto-oxidation in diabetes. Glucose auto-oxidation has been linked to non-enzymatic glycosylation, and glycosylated proteins provide a source of free radicals [55]. Oxidative stress in diabetes coincides with a decrease in the antioxidant status [56], which can increase the deleterious effects of free radicals. ROS-related oxidative stress plays an important role in the etiology of diabetic complications [57]. Various toxic substances produced by lipid peroxidation destroy surrounding tissues [58]. Moreover, increased lipid peroxidation in various organs was demonstrated in db/db mice, in which it acted as a potent redox cycler that generates harmful ROS and causes organ damage [59]. Oxidative stress induced by ROS can be eliminated by antioxidant enzymes. SOD rapidly converts the superoxide anion into hydrogen peroxide, which is degraded by CAT. GSH, a representative endogenous antioxidant, can prevent tissue damage by maintaining low levels and certain cellular concentrations of ROS [60]. In this study, we observed that 400 and 200 mg/kg fGT extract exerted stronger inhibitory effects on hepatic lipid peroxidation and depletion of endogenous antioxidant enzymes (SOD, CAT, and GSH) compared with GT extract. Thus, the decrease in ROS levels in fGT-treated db/db mice may be attributed to improved antioxidant capacity, which may contribute to reduced lipid peroxidation and protect against hepatic oxidative stress in type 2 diabetes.

## 5. Conclusions

fGT showed anti-obese, anti-diabetic hypoglycemic, anti-hyperlipidemia, and antioxidant effect in diabetic mice. In notice, fGT exerted stronger anti-diabetic activities compared with GT. In addition, 400 and 200 mg/kg fGT effectively attenuated the risk of nephropathies and hepatopathies compared with 400 mg/kg GT in db/db mice. These results suggested that fermentation with appropriated amounts of Aquilariae Lignum, 1:49 (2%), synergistically increased the anti-diabetic effects of GT in db/db mice. Thus, fGT will be promise as a new potent therapeutic agent for type 2 diabetes.
